# Data on analysis of MK-801 bioavailability in mouse plasma and brain tissue by ultra-performance liquid chromatography-tandem mass spectrometry

**DOI:** 10.1016/j.dib.2019.104623

**Published:** 2019-10-04

**Authors:** Baiba Svalbe, Solveiga Grinberga, Gundega Stelfa, Edijs Vavers, Baiba Zvejniece, Eduards Sevostjanovs, Maija Dambrova, Liga Zvejniece

**Affiliations:** a Latvian Institute of Organic Synthesis, Aizkraukles 21, Riga, LV-1006, Latvia; b Latvia University of Life Sciences and Technologies, K.Helmana 8, Jelgava, LV-3004, Latvia; c Riga Stradins University, Dzirciema 16, Riga, LV-1007, Latvia

**Keywords:** i.p. and s.c. administration route, MK-801, Bioavailability, Mice, UPLC-MS/MS

## Abstract

MK-801, a N-methyl-d-aspartate receptor antagonist, is widely used in animal preclinical experiments to induce memory and learning impairments and schizophrenia-like behavior. In the present study, we compared the plasma and brain tissue concentrations of MK-801 after intraperitoneal (i.p.) or subcutaneous (s.c.) administration at a dose of 0.1 mg/kg in male ICR mice. Moreover, these data present the optimization of ultra-performance liquid chromatography-tandem mass spectrometry (UPLC-MS/MS) for the analysis of MK-801 in biological samples. Procedures for the preparation of brain tissue and plasma samples and instrumental analysis are described. This article is related to a research article entitled “Effects of the N-methyl-d-aspartate receptor antagonist, MK-801, on spatial memory and influence of the route of administration” [1].

**Table:** Specifications Table

Subject area	Pharmacology
Specific subject area	Pharmacokinetics of MK-801 in mice
Type of data	Figures and table of analyzed data and chromatograms
How data were acquired	Ultra-performance liquid chromatography-tandem mass spectrometry (UPLC-MS/MS)
Data format	Raw and analyzed data
Parameters for data collection	Mouse plasma and brain samples were taken following subcutaneous and intraperitoneal injection of MK-801 at a dose of 0.1 mg/kg, purified on solid phase extraction (SPE), and injected into UPLC-MS/MS to determine drug concentration over time.
Description of data collection	Mouse brain homogenates were prepared. Blood was collected in heparin-coated tubes and centrifuged to obtain plasma. Plasma and brain homogenate samples were pretreated by solid-phase extraction using Strata-X reversed-phase cartridges. MK-801 concentration was determined by ultra-performance liquid chromatography-tandem mass spectrometry.
Data source location	Riga, Latvia
Data accessibility	Data in the article
Related research article	B. Svalbe, G. Stelfa, E. Vavers, B. Zvejniece, S. Grinberga, E. Sevostjanovs, O. Pugovics, M.Dambrova, L. Zvejniece Effects of the N-methyl-d-aspartate receptor antagonist, MK-801, on spatial memory and influence of the route of administration, Behav. Brain Res. 372, 2019, 112067, https://doi.org/10.1016/j.bbr.2019.112067[1]

**Table:** 

**Value of the data** 1. The data present concentrations of MK-801 in mouse brain tissue and plasma after s.c. and i.p. administration, which is important for experiment planning in experimental neuroscience. 2. These data will help researchers to choose the right administration route of MK-801 in the animal experiments. 3. The developed MK-801 quantification method (SPE and UPLC-MS/MS) can be used to determine MK-801 in mammalian tissue samples. 4. The data presented in this data article show that the concentration of MK-801 in brain tissues and plasma depend on the route of administration.

## 1 Data

The developed multiple reaction monitoring (MRM) method using SPE for sample pretreatment makes it possible to determine easily the MK-801 concentration in the mouse brain homogenate, unlike the Wegener method [2], which describes the determination of MK-801 in the brain after microdialysis, and a more complicated sampling method.

**Fig. 1:**
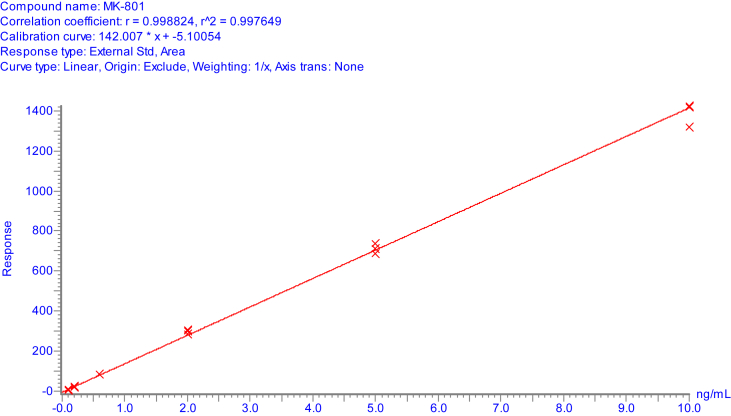
Calibration line of MK-801 in mouse brain tissue homogenate from 0.1 ng/mL to 10.0 ng/mL. LOQ = 0.20 ng/mL.

**Fig. 2:**
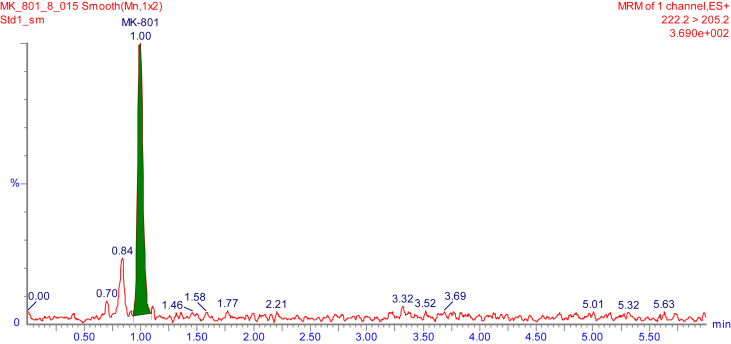
Representative MRM chromatogram of MK-801 in mouse brain tissue homogenate at LOQ (0.2 ng/mL).

**Fig. 3:**
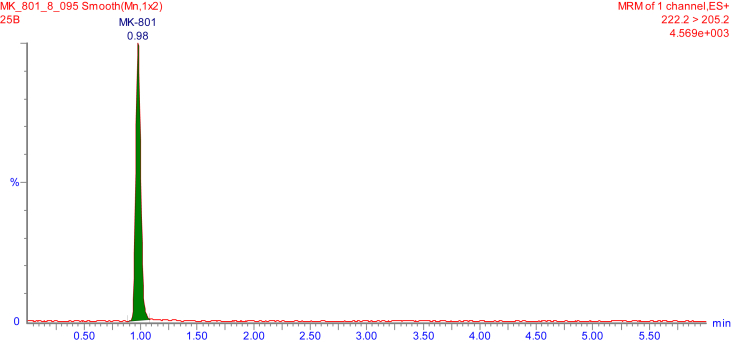
Representative MRM chromatogram of MK-801 in mouse brain tissue homogenate – A concentration of 1.79 ng/mL was found in the sample after 2 h of s.c. administration of MK-801 at a dose 0.1 mg/kg.

The data depicted in Fig. 1, Fig. 2, Fig. 3 show the calibration line obtained in mouse brain homogenates and representative MRM chromatograms of the calibration standard at the LOQ and a real mouse brain homogenate sample after MK-801 administration.

**Fig. 4:**
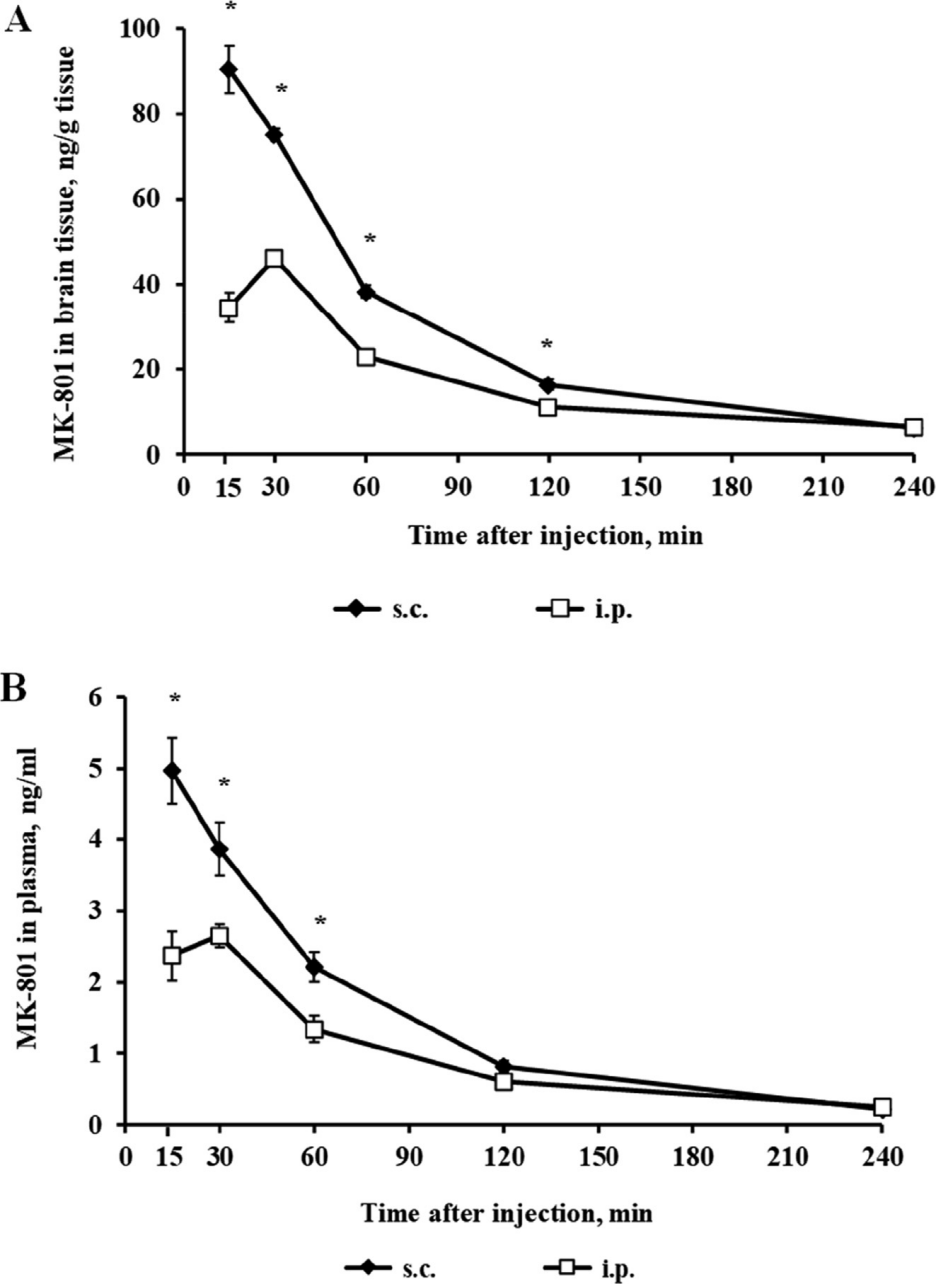
Concentrations of MK-801 in brain tissue and blood plasma. Mice received a s.c. or i.p. injection of MK-801 at a dose of 0.1 mg/kg. Brain tissue (A) and blood plasma (B) samples were collected 15, 30, 60, 120, and 240 min after MK-801 administration. Each point represents the mean (± SEM) of three mice per group. The data were analyzed for significant differences using unpaired Student's t-test. *p < 0.05 vs. the corresponding i.p. group.

The concentration of MK-801 was measured in blood plasma and brain tissue 15, 30, 60, 120, and 240 min after a single i.p. or s.c. administration of MK-801 at a dose of 0.1 mg/kg. The concentration of MK-801 in the brain was significantly higher after s.c. administration than after i.p. administration at all time points until the 120-min time point (Table 1; Fig. 4A).

**Table 1:** The concentration of MK-801 in blood plasma and brain tissue 15, 30, 60, and 120 min after a single i.p. or s.c. administration of MK-801 at a dose of 0.1 mg/kg. Corresponds to Fig. 4A and B.

Group	Brain tissue, ng/g					Blood plasma, ng/ml				
	Time (min)									
	15	30	60	120	240	15	30	60	120	240
**s.c.**	96.50	71.86	34.70	14.09	3.94	4.54	4.55	2.29	0.84	0.19
	93.24	76.26	33.41	15.08	6.30	5.87	3.29	1.83	0.95	0.31
	73.02	73.10	40.87	13.32	5.52	4.47	3.75	2.52	0.66	0.18
	106.64	80.21	42.64	16.99	6.61					
	98.41	71.87	38.85	21.92	7.97					
	74.56	77.62	39.68	17.24	6.34					
**Mean**	**90.39**	**75.15**	**38.36**	**16.44**	**6.11**	**4.96**	**3.87**	**2.21**	**0.82**	**0.22**
SEM	5.56	1.40	1.47	1.27	0.54	0.46	0.37	0.20	0.09	0.04
**i.p.**	41.18	44.10	17.20	11.95	6.20	2.10	2.69	0.98	0.79	0.24
	20.50	41.89	23.79	8.52	6.02	1.95	2.36	1.60	0.46	0.19
	37.49	47.57	24.91	7.98	6.97	3.06	2.90	1.45	0.59	0.30
	44.04	47.46	20.88	14.51	6.20					
	30.18	46.87	24.01	12.46	7.45					
	33.43	49.61	26.51	11.91	6.65					
**Mean**	**37.47**	**46.25**	**22.88**	**11.22**	**6.58**	**2.37**	**2.65**	**1.34**	**0.61**	**0.24**
SEM	3.47	1.13	1.36	1.02	0.22	0.35	0.16	0.19	0.10	0.03
**p**	*	*	*	*	**ns**	*	*	*	**ns**	**ns**

The bold indicates the most important data of the table, as it shows the mean. ns – non-significant; s.c. — subcutaneous; i.p. — intraperitoneal.

Identical effects were observed in blood plasma after MK-801 administration (Table 1; Fig. 4B). The concentration of MK-801 in blood plasma was significantly higher following s.c. injection than following i.p. administration until the 60-min (Fig. 4B). The maximum concentration was reached in brain tissue and blood plasma 15 min after s.c. injection and 30 min after i.p. administration of MK-801 at a dose of 0.1 mg/kg.

The maximal concentration (C_max_) of MK-801 in brain tissue and blood plasma were almost 2-fold higher when administered via the s.c. route than the i.p. route (Table 2). The concentration of MK-801 in brain tissue was approximately 59% higher after s.c. injection than after i.p. injection, as shown by the area under the curve (AUC, Table 2). The MK-801 AUC value for blood was over 1.5-fold higher after s.c. injection than after i.p. injection (Table 2).

**Table 2:** Pharmacokinetic parameters of MK-801 after s.c. or i.p. administration in brain tissue and blood plasma.

Administration route	Brain tissue		Blood plasma	
	C_max_ (ng/g)	AUC (ng min/g)	C_max_ (ng/ml)	AUC (ng min/ml)
**s.c.**, 0.1 mg/kg	90.4 ± 5.6	5940 ± 196*	5.0 ± 0.5	311 ± 8*
**i.p.**, 0.1 mg/kg	46.3 ± 1.1	3733 ± 123	2.7 ± 0.2	208 ± 9

C_max_ — maximal concentration; s.c. — subcutaneous; i.p. — intraperitoneal; AUC — area under the curve was calculated from 15 min to 240 min after drug administration. Each point represents the mean (± SEM) of three mice per group. The data were analyzed for significant differences using unpaired Student’s t-test. *p < 0.05 vs. the corresponding i.p. group.

## 2 Experimental design, materials and methods

### 2.1 Animals

All studies involving animals were conducted in accordance with ARRIVE guidelines [3,4]. Experimental procedures were performed in accordance with the guidelines reported in the EU Directive 2010/63/EU and local laws and policies. All procedures were approved by the Latvian Animal Protection Ethical Committee of Food and Veterinary Service in Riga, Latvia. Thirty male ICR mice weighing 25–30 g at the time of the pharmacokinetics experiment (Harlan Laboratories BV; Venray, Netherlands) were housed under standard conditions (21–23 °C, relative humidity of 65% ± 10%, 12-hour light-dark cycle) with unlimited access to standard food (R70 diet, Lactamin AB, Sweden) and water.

### 2.2 MK-801 administration

MK-801 ((+)-MK-801 hydrogen maleate) was dissolved in sterile saline (0.9% NaCl). MK-801 concentrations in the brain tissue and plasma were determined in mice receiving single i.p. or s.c. doses of MK-801 at 0.1 mg/kg 15 and 30 min, and 1, 2, and 4 hours before decapitation. Each group consisted of 3 animals for the retrieval of blood and brain samples. The brain was divided into two hemispheres, and 6 measurements were obtained per point.

### 2.3 Determination of MK-801 levels in blood plasma and brain tissue using UPLC-MS⁄MS

The concentrations of MK-801 in brain tissue and plasma were measured using UPLC-MS⁄MS. Blood was collected in heparin-coated tubes and centrifuged at 3000 rpm at 4 °C for 10 min to separate the plasma. The brain was gently removed and homogenized in ice-cold Milli-Q water at a w/v ratio of 1:5 in 20-second bursts using a Cole Parmer 130-Watt ultrasonic processor set to 40 kHz. The obtained homogenate was centrifuged at 16500 rpm for 10 min at 4 °C. The supernatant was then decanted, and the pellet was homogenized in the same volume of Milli-Q water as before. The obtained homogenate was centrifuged at 16500 rpm for 10 min at 4 °C. The supernatants were combined and stored at −80 °C until use.

Brain tissue extract (300 μL) were mixed with 700 μL of 2% formic acid aqueous solution (v/v), vortexed und centrifuged at 13000 rpm for 10 min. 100 μL of mouse plasma were mixed with 900 μL of 2% formic acid aqueous solution (v/v), vortexed and centrifuged at 13000 rpm for 10 min. 950 μL of the supernatant was load onto conditioned Strata X (33μm, 30mg, 1 mL) SPE cartridge (Phenomenex), washed 2 times with 1mL of 5% methanol aqueous solution and eluted with 900 μL of 1% formic acid solution in acetonitrile (v/v). The eluent was evaporated using a Genevac EZ-2 at 40 °C, reconstituted in 200 μL of mobile phase solution (acetonitrile/0.1% formic acid, 1:1, v/v), and subjected to UPLC-MS/MS analysis. Calibration was performed against external calibration standard solutions.

UPLC was carried out using the Waters Acquity UPLC system equipped with the Acquity BEH C8 column (2.1 × 75mm, 1.7 μm). The mobile phase consisted of A (aqueous 0.1% formic acid) and B (acetonitrile) at a flow rate of 0.25 mL/min, using a gradient elution: Initial – 50% B, 2.5 min–98% B, 4.5 min–98% B, 4.7 min −50% B, 6 min–50% B. The injection volume was 5 μL.

MS/MS analysis was performed on a Micromass Quattro MicroTM tandem mass spectrometer in positive ion mode using multiple reaction monitoring of the transition from *m*/*z* 222.2 to 205.2 (cone voltage 25V, collision energy 20eV). Quantitative analysis was achieved using QuanLynx 4.1 software (Waters, Milford, USA).

### 2.4 Chemicals

(+)-MK-801 hydrogen maleate (5S,10R)-(+)-5-Methyl-10,11-dihydro-5H-dibenzo [a,d]cyclohepten-5,10-imine hydrogen maleate) was purchased from Sigma Aldrich (St Louis, Missouri, USA). Ethanol was purchased from LAKO Ltd. (Riga, Latvia). 0.9% physiological saline was purchased from Fresenius Kabi (Warszawa, Poland). Acetonitrile and methanol were obtained from Merck (Darmstadt, Germany).

### 2.5 Statistical analysis

All data were expressed as the mean ± standard error of the mean (SEM). MK-801 concentrations in brain tissue and blood plasma were analyzed using unpaired Student's t-tests. P values less than 0.05 were significant. The statistical calculations were performed using the GraphPad Prism 3.0 software package (GraphPad Software, Inc., La Jolla, California, USA).
